# Orthodontic Treatment Needs in Children with Autism Spectrum Disorder

**DOI:** 10.3390/jcm14217743

**Published:** 2025-10-31

**Authors:** Magdalena Prynda, Wojciech Niemczyk, Agnieszka Anna Pawlik, Grzegorz Dawiec, Monika Dawiec, Beata Kazek, Mikołaj Mazur, Natalia Pschionko, Dariusz Skaba, Ewa Emich-Widera, Rafał Wiench

**Affiliations:** 1 M-Dent Center for Esthetic Dentistry and Implantology, 34a/7 Sienkiewicza St., 50-335 Wrocław, Poland; 2 Department of Dental Surgery, Faculty of Dentistry, Wroclaw Medical University, Krakowska 26, 50-425 Wroclaw, Poland; 3 Department of Periodontal Diseases and Oral Mucosa Diseases, Faculty of Medical Sciences in Zabrze, Medical University of Silesia, pl. Traugutta 2, 41-800 Zabrze, Poland; 4 Specialist Dental Clinic dr n.med. Agnieszka Anna Pawlik, ul. Strumieńskiego 12/4, 41-400 Mysłowice, Poland; 5 Department of Paediatric Otolaryngology, Head and Neck Surgery, Department of Paediatric Surgery, Faculty of Medical Sciences, ul. Medyków 16, 40-752 Katowice, Poland; 6 Outpatient Clinic for Dental Surgery in Zabrze, University Dental Centre, Silesian Medical University Ltd. in Katowice, pl. Akademicki 17, 41-902 Bytom, Poland; 7 Private Dental Practice NZOZ Stomatologia-Dawiec s.c., ul. Witczaka 49/15, 41-902 Bytom, Poland; 8 Department of Conservative Dentistry with Endodontics, Faculty of Medicine and Dentistry, Medical University of Silesia in katowice, pl. Akademicki 17, 41-902 Bytom, Poland; 9 Development Assistance Centre “CWR Persevere”, Kępowa 56 st., 40-583 Katowice, Poland; 10 Faculty of Medical Sciences in Zabrze, Medical University of Silesia, pl. Traugutta 2, 41-800 Zabrze, Poland; 11 Department of Child Neurology, Faculty of Medical Sciences, Medical University of Silesia, 40-752 Katowice, Poland

**Keywords:** autism spectrum disorder, child, cross-sectional studies, index of orthodontic treatment need, oral health, malocclusion

## Abstract

**Background/Objectives**: Autism spectrum disorder (ASD) is associated with a higher prevalence of oral health problems, including parafunctional habits and malocclusions, which may lead to increased orthodontic treatment needs. The objective of this study was to evaluate orthodontic disorders and treatment requirements in children with ASD compared to their neurotypical peers. **Methods**: A cross-sectional study was conducted on 148 children aged 3–12 years, including 74 children with ASD and 74 controls matched for age and sex. Data were collected via caregiver questionnaires and clinical dental examinations. Malocclusions and orthodontic treatment requirements were assessed using the Index of Orthodontic Treatment Need (IOTN), including both the Dental Health Component (DHC) and Aesthetic Component (AC). Statistical analyses included Mann–Whitney U tests, Student’s *t*-tests, and effect size calculations, with significance set at *p* ≤ 0.05. **Results**: Children with ASD exhibited significantly higher orthodontic treatment needs compared to controls, with elevated scores in both IOTN-DHC (*p* < 0.001) and IOTN-AC (*p* < 0.001). No significant differences were observed for the mean overjet or overbite between groups. Gender analysis revealed that boys with ASD had significantly higher scores in both IOTN-DHC and IOTN-AC, while girls with ASD differed from controls only in IOTN-AC. **Conclusions**: Children with ASD are at increased risk for orthodontic treatment, particularly for both health and aesthetic needs, with boys showing the most pronounced disparities. These findings highlight the importance of early orthodontic assessment and tailored preventive strategies in this population.

## 1. Introduction

Autism spectrum disorder (ASD) impacts an individual’s ability to both perceive and react to social information [1,2,3]. According to the extant literature, children diagnosed with ASD are more likely to exhibit signs of unusual tooth decay and even tooth loss when compared to their neurotypical peers. Furthermore, they are more prone to developing other dental health issues, including dental and soft-tissue traumas, as well as teeth grinding [2,4,5]. Within the context of oral health, individuals with ASD have a higher propensity for parafunctional oral habits, such as bruxism, tongue thrust, or nonnutritive chewing. These habits are recognized as indicators of malocclusion, including anterior open bite, posterior crossbite, and excessive overjet [6,7,8,9,10,11]. An increased overjet has been demonstrated to result in functional difficulties, including challenges related to biting into specific foods and maintaining optimal lip closure at rest [12,13,14,15]. Furthermore, ASD, as a neurodevelopmental disorder, has been associated with an elevated risk of speech impairments [16,17,18].

Malocclusion has been identified as the third most prevalent oral health problem in the general population, and it is regarded as a significant public health concern due to its high prevalence rate [19]. A multitude of studies have demonstrated that adolescents who have pursued orthodontic treatment, particularly those with more pronounced malocclusions, have exhibited a diminished quality of life with respect to oral health in comparison to those who have not sought orthodontic intervention [20,21].

As other researchers have noted, studies on malocclusions are limited, and there is a paucity of evidence regarding the complexity of malocclusions and the necessity of orthodontic treatment in children with ASD [22]. Consequently, the authors of this study have chosen to address this subject. The aim of this study was to assess whether there are differences in orthodontic disorders between children with ASD and their healthy peers. The null hypothesis is that there is no statistically significant difference in orthodontic treatment needs between children with autism spectrum disorder and neurotypical peers.

## 2. Materials and Methods

### 2.1. Study Design and Ethics

This research followed a cross-sectional approach, with data gathered between July 2019 and January 2022. Participation was contingent on obtaining written consent from each child’s parent or legal guardian. Ethical approval for the project was granted by the Bioethics Committee of the Silesian Medical University on 9 July 2019 (reference no. KNW/0022/KB1/82/19). The study adhered to the guidelines set forth in the Strengthening the Reporting of Observational Studies in Epidemiology (STROBE) checklist [23].

### 2.2. Study Participants

A total of 148 children aged 3–12 years participated in this cross-sectional study, including 74 with a diagnosis of autism spectrum disorder (ASD) and 74 neurotypical controls matched for age and sex.

Children with ASD were recruited through the Persevere Developmental Support Centre (CWR Persevere Niepubliczna Specjalistyczna Poradnia Psychologiczno-Pedagogiczna, Katowice, Poland), a facility specializing in diagnostic and therapeutic services for individuals on the autism spectrum. Eligible participants were identified by the center’s clinical staff based on prior diagnostic records and invited to participate after parental consent was obtained.

Control participants were recruited from local kindergartens and primary schools in the Silesian Province through posters and information leaflets. Parents or legal guardians voluntarily registered their children after receiving detailed information about the study and providing written informed consent.

All control participants underwent professional screening for neurodevelopmental disorders conducted by experienced specialists (B.K. and E.E.-W.) to ensure the absence of autism spectrum or related developmental conditions.

### 2.3. Inclusion and Exclusion Criteria

To ensure a homogeneous and representative sample, the same inclusion and exclusion criteria were applied consistently across the study.

Inclusion criteria:

- Age between 3 and 12 years;
- Written informed consent from a parent or legal guardian;
- For the study group: a confirmed diagnosis of autism spectrum disorder (childhood autism, atypical autism, or Asperger syndrome) established by a psychiatrist according to ICD-11 criteria;
- For the control group: absence of developmental, neurological, or chronic systemic conditions that could influence oral health or nutritional status.

Exclusion criteria:

- Age below 3 years or above 12 years;
- Presence of syndromic or secondary autism associated with intellectual disability, epilepsy, genetic syndromes, or congenital malformations;
- inability to complete the clinical assessment.

All eligible participants meeting these criteria were included in the final analysis.

### 2.4. Sample Size Calculation

The required sample size for comparing orthodontic treatment need between children with and without autism spectrum disorder was calculated using the following formula for two proportions [22,24]:

$$n=\;\frac{{\left(Z_{1-\alpha /2}+Z_{1-\beta }\right)}^{2}[p_{1}(1-p_{1})+\;p_{0}(1-p_{0})]}{{\left(p_{1}-p_{0}\right)}^{2}}$$

where $p_{0}$ is the proportion in the control group, $q_{0}$ = 1 − $p_{0}$, $p_{1}$ is the proportion in the ASD group, $q_{1}$ = 1 − $p_{1}$, ${\;Z}_{1-\alpha /2}$ is the standard normal deviate corresponding to a 95% confidence level (1.96), and ${\;Z}_{1-\beta }$ corresponds to 80% power (0.84).

Based on previously published data [22], the prevalence of orthodontic treatment need was $p_{0}$ = 0.449 in the control group and $p_{1}$ = 0.792 in the ASD group. Using these values, the calculation yielded a minimum sample size of 35 children per group.

$$n=\frac{{\left(1.96+0.84\right)}^{2}[(0.792)(0.208)+(0.449)(0.551)]}{{\left(0.792-0.049\right)}^{2}}\approx 34.7$$

*n* = sample size for study group

### 2.5. Data Collection

Data collection was conducted in two stages: a caregiver questionnaire and a standardized clinical examination, complemented by photographic documentation for orthodontic assessment.

#### 2.5.1. Questionnaire (Supplementary Material S1)

Parents or legal guardians completed a structured questionnaire covering perinatal history, feeding methods, oral hygiene habits, parafunctional behaviors (e.g., thumb sucking, bruxism, mouth breathing), and dental visit frequency. These variables were used to describe behavioral characteristics and explore potential associations with orthodontic findings. Items related to feeding or chewing patterns were collected to provide contextual information on oral function but were not included in the statistical analyses, as they fell outside the study’s primary objective of assessing orthodontic treatment needs.

#### 2.5.2. Clinical Examination

All children underwent a standardized intraoral and extraoral dental examination performed by two calibrated clinicians (M.P. and A.A.P.). The assessment focused on occlusal characteristics, presence of malocclusion traits, and indices required for the Index of Orthodontic Treatment Need (IOTN) [25]. Visual inspection was used to evaluate general craniofacial symmetry, facial proportions, and soft-tissue profile; no anthropometric measurements were performed. No participants exhibited abnormalities such as maxillofacial syndromes or hydrocephalus; thus, no exclusions were made on this basis.

Each examiner evaluated all participants independently, and a subset of 15 randomly selected children was re-examined after two weeks to assess intra- and inter-examiner consistency. Reliability was high (Cohen’s κ = 0.91 for intra-examiner and κ = 0.88 for inter-examiner agreement), indicating strong but concordant agreement.

#### 2.5.3. Photographic Documentation

Standardized extraoral and intraoral photographs were taken for diagnostic and analytical purposes.

Extraoral images documented facial proportions and symmetry (frontal at rest, frontal smiling, right and optional left profile) under neutral lighting against a gray background. A DSLR camera (manual mode, 85–105 mm lens) mounted on a tripod was used, positioned at the patient’s eye level and 2 m away, with exposure settings of f/9, 1/125–1/160 s, ISO 100–200, and ring flash illumination.

Intraoral images captured occlusal relationships (frontal, right and left lateral, upper and lower arches) using a 60–100 mm macro lens and ring flash, following AAO/EOS photographic standards (f/22–f/32, ISO 100–200).

All images were recorded in both RAW (for archiving) and JPEG (for analysis) formats. Camera calibration and consistent lighting ensured reproducible image quality.

#### 2.5.4. Data Management

Clinical and questionnaire data were recorded on standardized paper forms and later transcribed into a secure electronic database. Entries were cross-checked by two independent investigators to ensure accuracy and completeness before statistical analysis.

### 2.6. Index of Orthodontic Treatment Need (IOTN)

Dental Health Component (DHC):

The DHC was determined clinically by two trained examiners (M.P. and A.A.P.) using standard diagnostic instruments under artificial lighting. Each child’s malocclusion was graded on a five-point ordinal scale (grades 1–5), where grades 4–5 indicate a definite need for treatment and grade 3 represents a borderline need. The assessment included occlusal anomalies such as displacement, crossbite, overjet, open bite, and overbite severity, following official IOTN guidelines. Detailed evaluation criteria are presented in Supplementary Material S2.

Aesthetic Component (AC):

The AC was scored from standardized frontal intraoral photographs using the ten-point photographic reference scale accompanying the IOTN. Scores of 5–7 denote a borderline need, while 8–10 represent a definite aesthetic need for orthodontic intervention. Both examiners independently rated all images, blinded to group allocation and clinical findings.

Examiner calibration and reliability:

Prior to data collection, both examiners underwent a calibration session using a training set of 20 reference cases to standardize scoring criteria for both IOTN components. Agreement was tested on a separate subset of 15 participants assessed twice, two weeks apart.

Intra-examiner reliability was κ = 0.90 for DHC and κ = 0.88 for AC.

Inter-examiner reliability was κ = 0.86 for DHC and κ = 0.84 for AC.

These values reflect excellent agreement, confirming the consistency of evaluations across raters and time points.

Control for prior orthodontic treatment:

Before examination, caregivers were asked whether the child had ever worn orthodontic appliances or was currently undergoing orthodontic treatment. None of the participants in either group had received prior or ongoing orthodontic therapy; therefore, no data were excluded or adjusted for treatment history.

### 2.7. Statistical Analysis

All data were analyzed using STATISTICA version 12.0 (StatSoft Inc., Tulsa, OK, USA) and Microsoft Excel. The significance level was set at *p* ≤ 0.05.

Handling of missing data:

All questionnaires and examination forms were checked for completeness prior to data entry. Cases with incomplete key variables (e.g., missing IOTN scores or demographic data) were excluded from analysis. No imputation methods were applied because the proportion of missing data was <5% and distributed randomly across variables.

Descriptive statistics:

Quantitative variables were summarized as mean ± standard deviation (SD), median (Me), and interquartile range (Q1–Q3). Categorical variables were expressed as counts and percentages.

Assessment of data distribution and test selection:

The distribution of quantitative variables was evaluated using skewness values and visual inspection of histograms rather than formal normality tests such as Shapiro–Wilk, which are highly sensitive to small deviations in larger samples. Variables with approximately symmetrical distributions (absolute skewness < 1.0) were treated as normally distributed and analyzed using parametric tests. Variables showing skewed distributions or unequal sample sizes between comparison groups were analyzed with nonparametric tests.

Applied statistical tests:

- Student’s *t*-test was used for approximately normal quantitative variables with comparable variances (e.g., overjet and overbite).
- Mann–Whitney U test was applied for ordinal or non-normally distributed variables, including IOTN-DHC and IOTN-AC scores, and for subgroup comparisons by sex.
- Fisher’s exact test was used to assess differences in categorical variables such as oral habits and behavioral characteristics.
- Effect size measures were reported for significant results: Cohen’s d for *t*-tests, Glass’s rank biserial correlation (r(G)) for Mann–Whitney U tests, and the Yule coefficient (φ) for categorical variables.

No formal corrections were applied, as the analyses were hypothesis-driven and focused on a limited number of predefined comparisons.

## 3. Results

### 3.1. Demographic Characteristics

The study population comprised 148 Caucasian children, ranging in age from 3 to 12 years, with equal numbers in the study and control groups. The study group comprised 74 children diagnosed with ASD. The sample population was predominantly male, with 58 boys and 16 girls. The control group, which was selected using the same methodology, comprised 53 boys and 21 girls.

To ensure comparability of the results, baseline analyses were performed. A subsequent chi-square test of independence was conducted, confirming that the gender distribution between the ASD and control groups did not differ significantly. The statistical analysis employed for the evaluation of age disparities among groups and within gender subgroups involved the implementation of the Mann–Whitney U test. The results demonstrated a statistically significant difference in the ASD group, with boys exhibiting a higher mean age compared to girls (*p* < 0.001). No statistically significant disparities in age were observed between boys and girls in the control group, although boys were marginally older. Upon conducting a comprehensive comparison of the ASD and control groups, it was observed that there were no statistically significant disparities in terms of age or sex distribution. No statistically significant differences in age or sex distribution were observed between the ASD and control groups (*p* > 0.05); therefore, both groups were comparable in demographic characteristics (see Table 1).

Statistical analysis (Table 2) confirmed no significant differences between groups for either variable (*p* > 0.05). Within the ASD group, boys were significantly older than girls (*p* < 0.001). Therefore, both groups were considered homogeneous and comparable for subsequent analyses.

The difference in age between groups was not statistically significant. As our analyses focused on comparisons between groups rather than within groups, participants were not further divided by dentition stage, and all comparisons were conducted across the entire age range.

### 3.2. Variation in Overjet and Overbite and the Index of Orthodontic Treatment Needs

In the initial phase of the study, an investigation was conducted to ascertain whether subjects with and without ASD exhibited disparities in OVERJET, OVERBITE, IOTN-DHC, and IOTN-AC. To this purpose, Student’s *t*-test for independent samples was utilized (see Table 3).

The analysis showed statistically significant differences in IOTN-DHC and IOTN-AC scores. Participants with ASD had significantly higher mean orthodontic treatment needs scores for both aesthetic and health aspects. The difference was moderate for IOTN-DHC and large for IOTN-AC.

### 3.3. Differences in Overjet and Overbite and the Need for Orthodontic Treatment Between Groups Divided by Gender

To explore potential gender-related trends, orthodontic indices were described separately for boys and girls. However, because the female subgroups were small (n = 16 in ASD, n = 21 in controls), the statistical power for detecting meaningful differences was limited. Therefore, these analyses should be interpreted cautiously.

In boys, the mean IOTN-DHC and IOTN-AC scores tended to be higher in the ASD group compared to controls. In girls, the difference appeared less pronounced and was primarily related to aesthetic components. These gender patterns are presented descriptively in Table A1 and Table A2, without inferential interpretation.

### 3.4. Behavioral Characteristics

Table 4 presents an analysis of selected behaviors of children from both study groups. It is important to note the statistically significant difference in the frequency of independent tooth brushing by children in both groups. The difference was statistically significant (*p* < 0.05) with an almost moderate effect size. In addition, children with ASD are less likely to use dental floss. However, no statistically significant differences were found in chewing dysfunctions and parafunctions.

## 4. Discussion

### 4.1. General Outcomes

This study aimed to evaluate orthodontic disorders and treatment needs in children with ASD, comparing them with their neurotypical peers. The null hypothesis, which stated that there would be no significant difference between the two groups, was rejected. Children with ASD demonstrated markedly higher orthodontic treatment needs in both the IOTN-DHC and IOTN-ACs, while overjet and overbite measurements did not differ significantly. These results imply that malocclusions in ASD encompass more than just basic occlusal discrepancies and are influenced by broader functional and behavioral factors.

Despite the fact that a number of studies have examined the relationship between oral health and malocclusion in children diagnosed with ASD, the existing evidence remains inconsistent and frequently suffers from methodological limitations. A plethora of prior investigations have been hindered by the utilization of small, convenience samples or the absence of appropriate control groups, impeding the ability to draw definitive conclusions regarding the true prevalence and nature of orthodontic problems within this population. Furthermore, there is a paucity of studies that have comprehensively assessed both the dental health and aesthetic components of orthodontic treatment using standardized tools such as the IOTN. The present study, therefore, addresses this gap by employing a matched control design and validated assessment indices to provide a more robust evaluation of orthodontic treatment requirements in children with ASD. When compared with previous literature, our results align with reports by Meuffels et al. [22] and Fontaine-Sylvestre et al. [26] who also identified greater orthodontic treatment needs among children with ASD. However, some earlier studies, such as Farmani et al. [27], observed no significant group differences, possibly due to smaller, heterogeneous samples or differences in diagnostic criteria. Our use of a standardized assessment tool (IOTN) and matched control design strengthens the comparability of results and supports the conclusion that ASD is associated with increased orthodontic treatment requirements. Moreover, the observed gender-specific patterns—where boys exhibited higher health and aesthetic needs—add a new dimension rarely explored in prior research and warrant targeted preventive strategies.

These results align with previous reports highlighting the increased prevalence of malocclusions and orthodontic complications in children with ASD, suggesting that the disorder may predispose affected individuals to oral health problems requiring early and specialized orthodontic intervention [28].

A gender-based analysis yielded supplementary insights. A significant disparity was observed in the needs of boys with ASD across both the functional and aesthetic domains. In contrast, girls with ASD demonstrated discrepancies primarily in aesthetic considerations when compared to the control group. This gender-specific trend may be indicative of differences in oral habits, developmental trajectories, or behavioral patterns associated with ASD. These findings underscore the significance of customizing orthodontic assessment and treatment methodologies not only to the diagnosis of ASD but also to patient-specific factors, including gender.

The observed gender-related differences in the orthodontic treatment needs of children with ASD can be explained by a combination of biological, behavioral and developmental factors. Boys with ASD often exhibit greater symptom severity and behavioral rigidity, which can manifest as stronger parafunctional habits, such as bruxism, tongue thrusting or mouth breathing. These factors are known to contribute to malocclusion. Previous studies have shown that boys with ASD tend to demonstrate more pronounced motor stereotypies and oral self-stimulatory behaviors, which may influence dental arch development and occlusal relationships [29].

In contrast, girls with ASD typically display milder behavioral symptoms and greater social adaptability, which may reduce the occurrence of mechanically harmful oral habits. This could explain why significant differences in our study were limited primarily to aesthetic components. Additionally, hormonal and growth-related differences between genders during early childhood could influence craniofacial development and orthodontic patterns, further contributing to these disparities [30,31,32].

From a clinical perspective, these findings suggest that gender-specific behavioral and developmental factors should be considered when planning orthodontic care for children with ASD. Early behavioral modification and preventive strategies may be particularly beneficial for boys, who appear to be at a higher risk of functional and aesthetic orthodontic complications.

The extant literature on the prevalence of malocclusion in children with ASD has yielded contradictory results. A preponderance of research has previously indicated a heightened occurrence of malocclusions among children diagnosed with ASD [26,33]. While other researchers did not observe a significant difference, they did demonstrate that specific malocclusion traits are more prevalent in individuals with ASD [27,34,35].

The findings of this study are consistent with previous reports indicating that children with ASD are more likely to exhibit parafunctional oral behaviors, such as bruxism, tongue thrusting, or nonnutritive chewing, which can contribute to malocclusion [26,36,37]. Children diagnosed with ASD frequently exhibit heightened sensitivity in the oral region, manifesting as pronounced aversive reactions to tactile stimulation and diverse textures of foodstuffs or objects placed within this domain [38]. The prevalence of food selectivity among children with ASD ranges from 46 to 89% [39]. Dietary preferences, frequently constrained to soft and cariogenic foods due to sensory sensitivities, may also contribute to alterations in normal dental development and occlusal relationships [22]. Furthermore, cognitive, motor, and behavioral challenges commonly associated with ASD may contribute to higher susceptibility to oral trauma and irregularities in dental eruption patterns, further compounding orthodontic problems [40,41,42,43].

The elevated orthodontic needs observed in this population also carry psychosocial implications. Malocclusions, particularly those affecting dental esthetics, have been demonstrated to exert a detrimental influence on various domains of functioning in children and adolescents, including self-esteem, social interactions, and quality of life [44,45]. For children diagnosed with ASD, who already encounter considerable challenges in social communication, additional aesthetic concerns have the potential to exacerbate difficulties in peer relationships and emotional well-being. This underscores the necessity for early orthodontic screening and intervention, not only for functional benefits but also to support psychosocial development [12].

From a clinical perspective, these findings underscore the significance of integrating orthodontic assessment into routine dental care for children with ASD. Preventive strategies should be emphasized, including the early identification and correction of harmful oral habits, dietary counseling, and the reinforcement of oral hygiene practices adapted to the child’s sensory and behavioral profile. Furthermore, orthodontists and pediatric dentists must be prepared to implement individualized treatment approaches that accommodate the unique behavioral and sensory sensitivities of children with ASD. This often requires modified communication strategies, desensitization protocols, and multidisciplinary collaboration with caregivers and therapists [46].

### 4.2. Limitations

Several methodological limitations should be acknowledged. The cross-sectional design precludes causal inference. Convenience sampling and recruitment from a single center may introduce selection bias and limit the study’s generalizability. Furthermore, although the groups were matched for age and sex, potential confounders such as socioeconomic status, dietary habits and access to dental care were not controlled for, and these factors may have influenced the need for treatment. Lastly, the relatively homogeneous sample of Polish children restricts extrapolation to broader populations. Additionally, gender subgroup analyses were underpowered, particularly among girls (n = 16 in ASD, n = 21 in controls). Post hoc power evaluation indicates that even large effect sizes would have less than 50% power to be detected in these subgroups. Furthermore, the sample size calculation was performed for the overall ASD vs. control comparison, not for stratified analyses. Therefore, gender-related findings should be interpreted as exploratory, and future studies with larger, stratified samples are needed to confirm these trends. Future studies should adopt multicenter and longitudinal designs to confirm these findings and clarify the behavioral and biological mechanisms underlying orthodontic disparities in ASD.

### 4.3. Clinical Implications

The results of this study underscore the imperative for adapting orthodontic interventions to the unique requirements of children diagnosed with ASD. The incorporation of preliminary screening for malocclusions into routine dental visits is a crucial aspect of dental care, as it facilitates the timely identification of emerging orthodontic concerns. Preventive strategies, such as discouraging parafunctional oral habits, providing dietary guidance, and reinforcing tailored oral hygiene routines, are of particular importance in this group.

When orthodontic treatment is indicated, clinicians must consider the potential challenges related to sensory sensitivities, communication barriers, and behavioral rigidity. The implementation of customized desensitization protocols, visual aids, and structured behavioral reinforcement has been demonstrated to enhance patient cooperation and treatment outcomes [47]. The integration of expertise from various disciplines, such as orthodontics, pediatric dentistry, speech therapy, and behavioral science, may further enhance the efficacy of treatment outcomes. A patient-centered, multidisciplinary approach is imperative to ensure that children with ASD receive equitable, effective, and compassionate orthodontic care.

## 5. Conclusions

This study demonstrates that children with ASD require orthodontic treatment far more often than other children, particularly boys. This highlights the importance of early, personalized and preventive care. These findings highlight the need to integrate orthodontic assessment into routine dental care for children with ASD, supported by tailored behavioral and sensory-sensitive management strategies. Future longitudinal and interventional studies should explore the causal mechanisms involved and evaluate the effectiveness of early preventive and behavioral interventions in reducing orthodontic complications in this population.

## Figures and Tables

**Table 1:** Characteristics of the study groups.

Variable	ASD Group (n = 74)	Control Group (n = 74)	Total (n = 148)
Sex			
Female, n (%)	16 (21.6%)	21 (28.4%)	37 (25.0%)
Male, n (%)	58 (78.4%)	53 (71.6%)	111 (75.0%)
Age (years)			
Mean ± SD	9.0 ± 2.2	8.5 ± 2.6	8.7 ± 2.4
Median [Q1–Q3]	9.0 [7.0–11.0]	8.0 [6.0–11.0]	—

SD—standard deviation; Q1—lower quartile; Q3—upper quartile.

**Table 2:** Statistical comparison of demographic characteristics between groups.

Comparison	Test Used	*p*-Value	Interpretation
Sex distribution (ASD vs. Control)	χ^2^ test with Yates correction	0.45	NS (no difference)
Age (years)	Mann–Whitney U test	0.18	NS (no difference)
Age: ASD boys vs. ASD girls	Mann–Whitney U test	<0.001	Significant (boys older)
Age: Control boys vs. Control girls	Mann–Whitney U test	0.054	NS (trend)

**Table 3:** Comparison of overjet, overbite, and IOTN scores between ASD and control groups.

	ASD (n = 74)		Control Group (n = 74)				
Variable	M	SD	M	SD	*t*	*p*	*d* Cohen
Overjet	2.35	0.97	2.12	0.76	1.60 ᵃ	0.111	0.26
Overbite	1.96	1.04	1.93	0.80	0.18 ᵃ	0.860	0.03
IOTN-DHC	3.51	1.33	2.58	1.46	4.06	**<0.001**	0.67
IOTN-AC	6.65	2.39	4.30	2.38	6.00	**<0.001**	0.99

n—number of observations; M—mean; SD—standard deviation; *t*—test statistic value; *p*—statistical significance. ^a^ Levene’s test result was statistically significant—the result was reported with Welch’s correction.

**Table 4:** Parafunctional habits and oral hygiene behaviors in children with and without ASD.

Behavior	ASD Group (n = 74; 100%)	Control Group (n = 74; 100%)	Fisher’s Exact Test (*p*-Value)
pacifier sucking for calming purposes	42 (56.8%)	32 (43.2%)	*p* = 0.069
thumb sucking	13 (17.6%)	14 (18.9%)	*p* = 1.00
nail biting	29 (39.2%)	30 (40.5%)	*p* = 1.00
teeth grinding	33 (44.6%)	37 (50.0%)	*p* = 0.62
pencil biting	34 (46.0%)	32 (43.2%)	*p* = 0.87
mouth breathing/mixed breathing	47 (65.5%)	42 (56.8%)	*p* = 0.50
chewing hard foods	55 (74.3%)	55 (74.3%)	
brushing teeth independently	69 (93.2%)	58 (78.4%)	***p* < 0.05;** **(ES) ϕ = 0.21**
using dental floss	2 (2.7%)	19 (17.6%)	***p* < 0.01;** **(ES) ϕ = 0.25**

Note: Fisher’s exact test (two-tailed); Yule’s ϕ used as effect-size measure. Values > 0.20 ≈ small-to-moderate effect).

## Data Availability

The raw data supporting the conclusions in this article will be made available by the authors on request.

## Supplementary Materials

The following supporting information can be downloaded at https://www.mdpi.com/article/10.3390/jcm14217743/s1. Supplementary Material S1: Complete questionnaire for parents of patients; Supplementary Material S2: Precise methodology for assessing the IOTN index.

## Appendix A

**Table A1:** Comparison of means for overjet and overbite and IOTN between girls in both groups.

	ASD (n = 16)		Control Group (n = 21)				
Variable	M	SD	M	SD	*t*	*p*	*d* Cohen
Overjet	2.50	0.97	2.24	0.83	0.89	0.382	0.29
Overbite	2.06	1.12	1.67	0.66	1.34	0.188	0.45
IOTN-DHC	3.56	1.09	3.14	1.24	1.07	0.290	0.36
IOTN-AC	6.81	2.04	5.29	1.82	2.40	**0.022**	0.80

n—number of observations; M—mean; SD—standard deviation; *t*—test statistic value; *p*—statistical significance.

**Table A2:** Comparison of means for overjet and overbite and IOTN between boys in both groups.

	ASD (*n* = 58)		Control Group (*n* = 53)				
Variable	M	SD	M	SD	*t*	*p*	*d* Cohen
Overjet	2.31	0.98	2.08	0.73	1.44 ᵃ	0.152	0.27
Overbite	1.93	1.02	2.04	0.83	−0.61 ᵃ	0.546	0.11
IOTN-DHC	3.50	1.39	2.36	1.49	4.17	**<0.001**	0.79
IOTN-AC	6.60	2.49	3.91	2.47	5.72	**<0.001**	1.09

n—number of observations; M—mean; SD—standard deviation; *t*—test statistic value; *p*—statistical significance. ᵃ Levene’s test result was statistically significant—the result was reported with Welch’s correction.
