# Supplementary material for: Orthodontic Treatment Needs in Children with Autism Spectrum Disorder
Source: J Clin Med. 2025 Oct 31;14(21):7743. doi: 10.3390/jcm14217743 (PMC12610464; doi:10.3390/jcm14217743)
Supplement: Supplementary file 1 [file jcm-14-07743-s001.zip › jcm-3945607-supplementary.pdf]

Supplementary Material 1

Dental interview
(please circle the correct answer or write it in the space provided)

1. The child was born
a. naturally / by C-section
b. in which week of pregnancy
2. Feeding
a. natural until……………….
b. bottle-fed until...
3. Appearance of
a. first milk tooth (at what month of age...)
b. permanent tooth year (what month/year...)
4. Habits:
Thumb sucking YES/NO
Nail biting YES/NO
Teeth grinding YES/NO
Pacifier or bottle (for calming) YES/NO
until when……..
Pencil biting YES/NO
Other …………………………..
5. Breathing
a. Through the mouth YES/NO
b. Through the nose YES/NO
6. Chewing hard foods (e.g., carrots, apples) YES/NO
7. First words (month/year)
8. Oral hygiene and cleaning
Does the child brush their teeth themselves YES/NO
a. How many times a day ……..
b. When: before meals/after meals
Manual/electric toothbrush
Water flosser YES/NO how often……..
Mouthwash YES/NO How often…….
Dental floss YES/NO How often
9. Has the child had their first dental appointment yet?
If so, when (please provide the approximate date
…….
10. Is the child under regular dental supervision YES/NO
How often are the visits ………………………………………………………
What is the reason for the visits…………….
Does the child wear braces YES/NO
what kind……………………..

…….
11. Are there check-ups? YES/NO
Preventive care? YES/NO
Teeth sealing? YES/NO How many?.........

11. What dental procedures, including orthodontic procedures, have been performed so far?
12. Was there fluoride prevention?

Supplementary Material 2

**Grade 1 – No treatment required**	
Overjet	Normal (<3.5mm)
Reverse Overjet	Absent
Overbite	Normal (<3.5mm)
Openbite	Normal (<1mm)
Crossbite	Absent
**Grade 2 – Slight need for treatment**	
Overjet	>3.5mm but <=6 mm with competent lips
Reverse Overjet	Present but <=1mm
Overbite	>3.5mm without gingival contact
Openbite	>1mm but <=2mm
Crossbite	Mono- or bi-lateral with <=1mm discrepancy
**Grade 3 - Borderline**	
Overjet	>3.5mm but <=6 mm with incompetent lips
Reverse Overjet	>1mm but <=3.5mm
Overbite	>3.5mm without gingival or palatal trauma
Openbite	>2mm but <=4mm
Crossbite	Mono- or bi-lateral with >1mm but <=2mm discrepancy
**Grade 4 – Treatment required**	
Overjet	>6mm but <=9mm
Reverse Overjet	>3.5mm with no masticatory or speech difficulties<br>OR<br>>1mm but <=3.5mm with masticatory or speech difficulties
Overbite	>3.5mm with gingival or palatal trauma
Openbite	>4mm
Crossbite	Mono- or bi-lateral with >2mm discrepancy
**Grade 5 – Treatment required**	
Overjet	>9mm
Reverse Overjet	>3.5mm with masticatory or speech difficulties
